# Prediction model for aneuploidy in early human embryo development revealed by single-cell analysis

**DOI:** 10.1038/ncomms8601

**Published:** 2015-07-07

**Authors:** Maria Vera-Rodriguez, Shawn L. Chavez, Carmen Rubio, Renee A. Reijo Pera, Carlos Simon

**Affiliations:** 1 Institute for Stem Cell Biology and Regenerative Medicine, Center for Reproductive and Stem Cell Biology, Stanford, California 94305, USA.; 2 Department of Obstetrics and Gynecology, Institute for Stem Cell Biology and Regenerative Medicine, Center for Reproductive and Stem Cell Biology, Stanford, California 94305, USA.; 3 IGenomix, Parc Cientific Universitat de Valencia, Catedratico Agustin Escardino 9, Paterna 46980, Valencia, Spain.; 4 Department of Obstetrics and Gynecology, Fundación Instituto Valenciano de Infertilidad, University of Valencia, INCLIVA Health Research Institute, Paterna 46980, Valencia, Spain.

## Abstract

Aneuploidies are prevalent in the human embryo and impair proper development, leading to cell cycle arrest. Recent advances in imaging and molecular and genetic analyses are postulated as promising strategies to unveil the mechanisms involved in aneuploidy generation. Here we combine time-lapse, complete chromosomal assessment and single-cell RT–qPCR to simultaneously obtain information from all cells that compose a human embryo until the approximately eight-cell stage (*n*=85). Our data indicate that the chromosomal status of aneuploid embryos (*n*=26), including those that are mosaic (*n*=3), correlates with significant differences in the duration of the first mitotic phase when compared with euploid embryos (*n*=28). Moreover, gene expression profiling suggests that a subset of genes is differentially expressed in aneuploid embryos during the first 30 h of development. Thus, we propose that the chromosomal fate of an embryo is likely determined as early as the pronuclear stage and may be predicted by a 12-gene transcriptomic signature.

## Abstract (web summary)

Aneuploidy may be fatal for the embryo, hence predicting its occurrence is important for successful *in vitro* fertilization. Here the authors monitor development of human preimplantation embryos in real-time and correlate the blastomere ploidy with cleavage dynamics and gene expression, identifying 12-transcript signature that determines ploidy.

## Graphical abstract

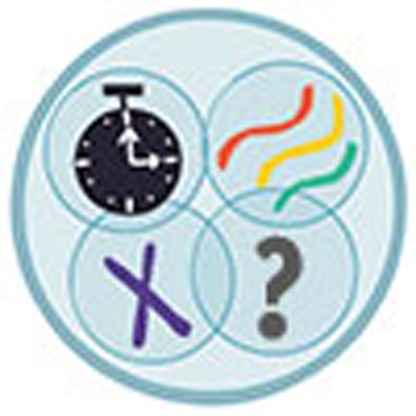

Over the last 20 years, the number of assisted reproduction procedures has drastically increased and is expected to continue to do so as parenthood is postponed. According to European statistics, more than half a million *in vitro* fertilization (IVF) cycles are performed annually, resulting in 100,000 newborns or 1.5% of all babies born in Europe^1^. Concomitant with the increased use of assisted reproduction, there is an increase in the number of studies that have investigated the dynamics of human embryo development. Time-lapse imaging provides a non-invasive alternative to the static morphological assessment of embryos, allowing the evaluation of embryo viability via the measurement of predictive parameters on the basis of developmental kinetics^2^,^3^. In addition, time-lapse allows the observation of cellular events that would otherwise remain undetected by conventional methods such as bipolar one- to three-cell divisions^4^,^5^, fragment reabsorption^6^,^7^ and blastomere fusion^5^. Technologies such as array comparative genomic hybridization (aCGH) have facilitated the transition from the study of nine chromosomes using fluorescent *in situ* hybridization (FISH) to the analysis of all 23 chromosome pairs simultaneously in a single cell^8^. More recently, several studies have combined additional technologies in order to generate a ploidy prediction model on the basis of embryo kinetics^7^,^9^,^10^.

Recent advances in the analysis of gene expression at the single-cell level provide the opportunity to examine underlying molecular programmes involved in embryo ploidy generation. Owing to technical limitations, previous studies of quantitative reverse transcription PCR (RT–qPCR) of human embryo development analysed only a select group of genes^11^,^12^ and/or large pools of embryos^13^, which can be confounded by potential embryo heterogeneity. As RT–qPCR techniques have evolved and become more sensitive, gene expression analysis of individual human embryos followed^3^,^14^ and, more recently, single-cell RT–qPCR analysis has become a reality^3^,^15^,^16^. To date, only a couple of studies have correlated gene expression patterns with aneuploidy in human embryos, one of which observed differential expression of certain epigenetic mediators in euploid versus aneuploid embryos and the other examining DNA repair genes in embryos with single over complex aneuploidies^17^,^18^. However, the latter study did not include euploid embryos, only evaluated six chromosomes via FISH and analysed 15–20 pooled day-4 embryos rather than individual embryos or single cells.

Here we seek to examine the relationship between aneuploidy, embryo kinetics and transcriptome profiles at the earliest stages of the human preimplantation development. By simultaneously assessing imaging behaviour, complete chromosomal composition and the expression of ∼90 genes in single cells from whole human embryos cultured at the one- to approximately eight-cell stage, we test the hypothesis that generation of aneuploidy may influence or be influenced by changes in gene expression and embryo kinetics. Our results show that aneuploid embryos exhibit altered developmental timing and a different transcriptomic profile than euploid embryos within the first 30 h of development. Thus, future studies should focus on the zygote as a key stage of human embryonic development and a potential source of non-invasive biomarkers that can prospectively predict ploidy.

## Results

### Early human embryo developmental kinetics and morphology

**Figure 1:**
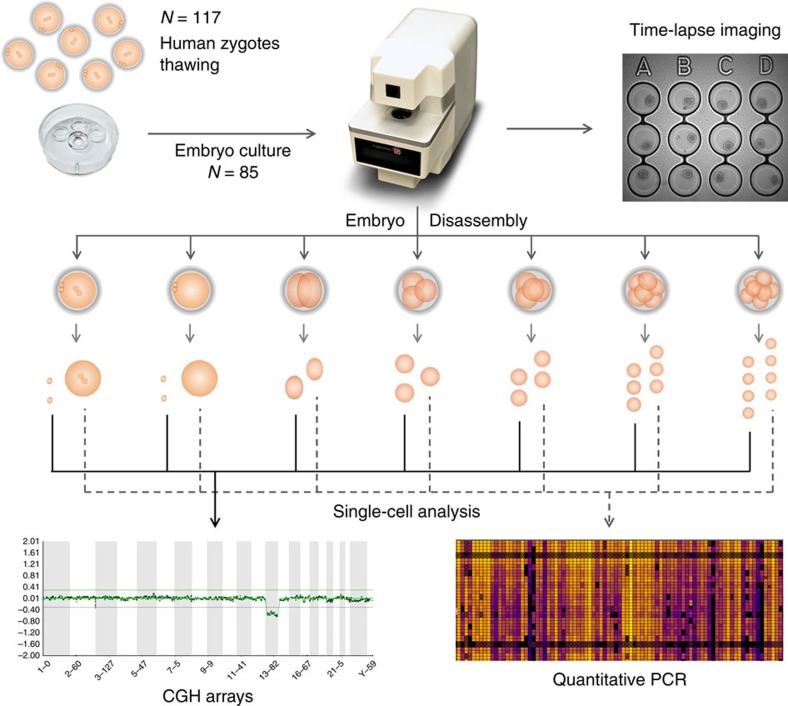
Experimental design of the study. One-hundred seventeen human embryos at the zygote stage were thawed, eighty-five of them survived and were cultured in nine different experiments. Embryo culture was performed in alphanumeric-labelled Petri dishes to allow embryo tracking during time-lapse imaging. Embryos were removed at different times until approximately the eight-cell stage. The number of cells varied depending on the type of divisions: one to two, one to three or one to four. Embryos were disaggregated into individual cells. Half of the cells from each embryo were analysed using aCGH to determine the ploidy status and the other half were analysed using RT–qPCR to study gene expression. Time-lapse movies were generated for each embryo and kinetic parameters were analysed.

Eighty-five cryopreserved human zygotes were cultured for different time periods ranging from 2.3 to 64.9 h (Fig. 1). Developmental kinetics of each embryo were translated from frames to hours on the basis that an image frame was captured every 5 min. All timing intervals between the one- and nine-cell stages were measured for each embryo unless removed for molecular or chromosomal analysis before reaching this stage of development. We began by examining previously reported parameters including the duration of the first cytokinesis, the time between the two- and three-cell, and time between the three- and four-cell stages^2^,^3^ (Supplementary Table 1). The median duration of the first cytokinesis was 20 min (range 15 min to 2.9 h); the time from two- to three-cell stage was 11.4 h (range 0–16.8 h); and the median time between the three- and four-cell stage was 1.3 h (range 0–22.1 h). Besides evaluating previously identified imaging parameters, we also measured the time between pronuclei disappearance (PNd) and the start of the first cytokinesis, a recently described parameter that has been linked to human embryo viability^4^,^5^, but not to the chromosomal status. As shown in Supplementary Table 1, the median for the time between PNd and the start of the first cytokinesis was 2.7 h (range 15 min to 22.2 h), suggesting that the wide range of this parameter might reflect underlying differences in embryo developmental potential.

In addition to normal cell cycle divisions, we also were able to detect abnormal division events in certain embryos, including divisions from one to three cells or from one to four cells instead of the typical one to two cells. These atypical events were observed in 23.6% (*n*=20) of embryos with the majority dividing from one to three blastomeres (*n*=17; Supplementary Movie 1) and a much smaller subset dividing from one to four cells (*n*=3; Supplementary Movie 2). Notably, 85% (*n*=17) of all abnormal divisions occurred during the first mitosis, whereas only 15% (*n*=3) occurred in either the second or third mitosis (Supplementary Movie 3), stressing the importance of the first mitotic division. Since irregular divisions produce a greater number of fast-dividing cells, the number of blastomeres was disregarded as a method to stage the embryos. As an example, an embryo with four blastomeres typically results from three consecutive normal divisions; however, it could also be produced during one direct cleavage from one to four cells. In both cases, the embryos would be at the four-cell stage and it would be inappropriate to similarly classify them. Thus, the time from PNd was chosen as the starting reference point to define the embryo stage, since it was the first event observed post thaw and before mitotic divisions.

With regards to morphology, we also evaluated the incidence and timing of cellular fragmentation using multiplane imaging in order to obtain a three-dimensional model of each embryo and an accurate measurement of fragmentation degree. The vast majority of embryos exhibited less than 25% fragmentation (*n*=62), and no embryo showed fragmentation greater than 60% (Supplementary Fig. 1a). Of the 64 embryos displaying fragmentation to any degree, most of them (68.8%) initially fragmented during the first division, 21.9% of embryos fragmented before and 9.4% after the completion of this division, further highlighting the importance of the first mitosis (Supplementary Fig. 1b).

### Correlation between the ploidy status and developmental kinetics

**Figure 2:**
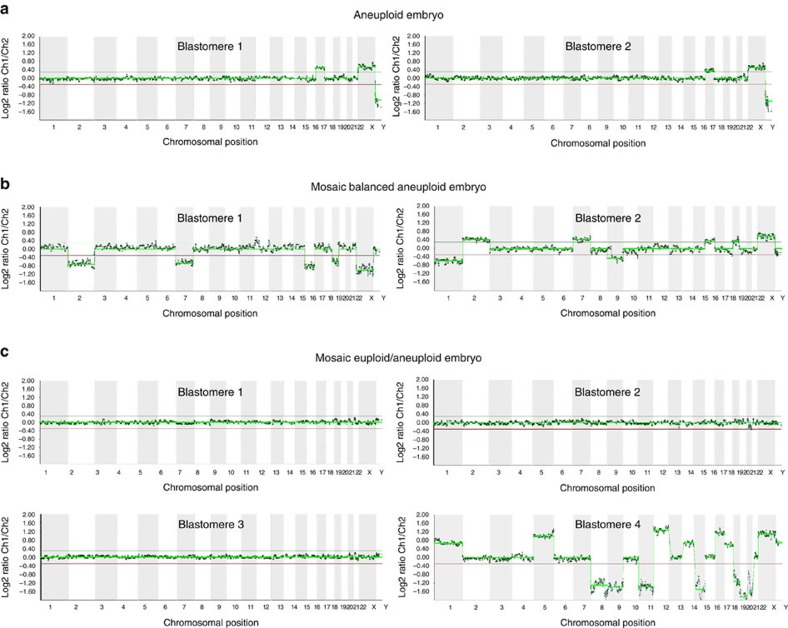
Representative aCGH results. (**a**) Two blastomeres from the same four-cell embryo showing chromosome 17 trisomy. (**b**) Two blastomeres from a chromosomally mosaic four-cell embryo with balanced aneuploidies for chromosomes 2, 7, 16, 19 and the sex chromosomes (Y0 and XXY). (**c**) Four blastomeres from a mosaic eight-cell embryo with three euploid blastomeres (46 XY) and one blastomere with multiple aneuploidies. All profiles were compared with the male control DNA reference.

Complete ploidy results were obtained for 89 cells from a total of 57 embryos. The incidence of aneuploidy was 50.9% (*n*=29) and in agreement with previous reports^7^,^19^,^20^. In particular, 23 embryos were diagnosed with defined aneuploidies, 8 of them with a single chromosomal abnormality (Fig. 2a) and 15 with more than one chromosome affected. Interestingly, six embryos displayed mosaicism among blastomeres: in three embryos, all blastomeres were chromosomally abnormal but with different abnormalities and complementary in some cases (Fig. 2b), whereas three embryos exhibited a mixture of euploid and aneuploid blastomeres (Fig. 2c).

We next evaluated developmental kinetics of aneuploid versus euploid embryos to determine which parameter(s) may be correlated with ploidy. Only two imaging parameters were statistically significant between aneuploid and euploid embryos when measured individually (Table 1). However, since embryos were taken out for analysis at different developmental times, it is important to note that the sample size gradually decreased as development proceeded, making the analysis of statistically significant differences for later parameters difficult. The most significant parameter was the time between the PNd and the start of the first cytokinesis (*P*=0.025, Mann–Whitney *U*-test), which was longer in aneuploid embryos compared with euploid embryos. As Table 1 demonstrates, the time between the three- and four-cell stage was also statistically different between aneuploid and euploid embryos (*P=*0.048, Mann–Whitney *U*-test) and approximately three times longer in aneuploid embryos. We also observed that the number of embryos with abnormal divisions in the aneuploid group was considerably higher (12/26) when compared with the euploid group (4/22). To determine whether there were differences in the ploidy status owing to this phenomenon, we analysed the developmental kinetics of normal versus abnormal embryo divisions separately (Table 1), since abnormal divisions directly influence parameter timing. When we separated the embryos solely based on normal and abnormal divisions, we did not detect a significant difference in the timing from the three- to four-cell stage between euploid and aneuploid embryos to confirm that abnormal divisions at this stage of development may reflect overall embryos' ploidy status. Nevertheless, we detected statistically significant differences in the time between PNd and start of the first cytokinesis between ploidy groups in embryos with normal divisions (*P*=0.049, Mann–Whitney *U*-test), but not in embryos with abnormal divisions. Surprisingly, 4 of 20 embryos that underwent irregular divisions also exhibited normal ploidy results. Three of these embryos were analysed immediately after the first mitosis—the irregular one—and only one blastomere from each embryo was analysed using aCGH. The fourth embryo also exhibited an abnormal first division but was removed for analysis at the nine-cell stage and the three blastomeres examined using aCGH were male euploid. No multinucleation was detected in any of the four embryos and, notably, three of the four embryos were from the same couple indicating an individual dimension of embryo development.

We next determined whether there was a correlation between the embryo ploidy status and the incidence of fragmentation on the basis of previous observations up to the four-cell stage^7^. For this purpose, we examined whether there was an association between aneuploidy and fragmentation degree in embryos with low (<25%) versus high (≥25%) cellular fragmentation. While 46.3% of the embryos with low fragmentation were aneuploid (19/41), the incidence in aneuploidy was 62.5% (10/16) in highly fragmented embryos. This observation was not statistically significant and suggests that the measurement of fragmentation degree alone is not predictive of the embryo ploidy status.

### Assessment of single-blastomere gene expression profiles

Single-cell gene expression results were obtained for 119 blastomeres from 78 embryos. A total of 87 genes were selected on the basis of their previously reported importance in the literature^3^,^21^,^22^,^23^,^24^,^25^,^26^,^27^,^28^,^29^,^30^,^31^,^32^,^33^,^34^,^35^. The biological processes in which the genes were involved included, but not limited to, cell cycle regulation, apoptosis, telomere maintenance and DNA methylation. Individual gene expression patterns were analysed in individual blastomeres to determine the progression of expression levels during preimplantation development. To compare embryos at different stages, PNd was designated as zero in the timescale and 56 h afterwards was set as the final time point since no gene expression data were obtained beyond this. In order to create a ‘best fit' model that allowed the identification of statistically different gene profiles in embryos, a quadratic regression was performed for each gene as described in Methods. Using this regression, we observed statistically significant differences in 55 of the 87 genes analysed [analysis of variance (ANOVA) test, *P*<0.05; Supplementary Table 2].

**Figure 3:**
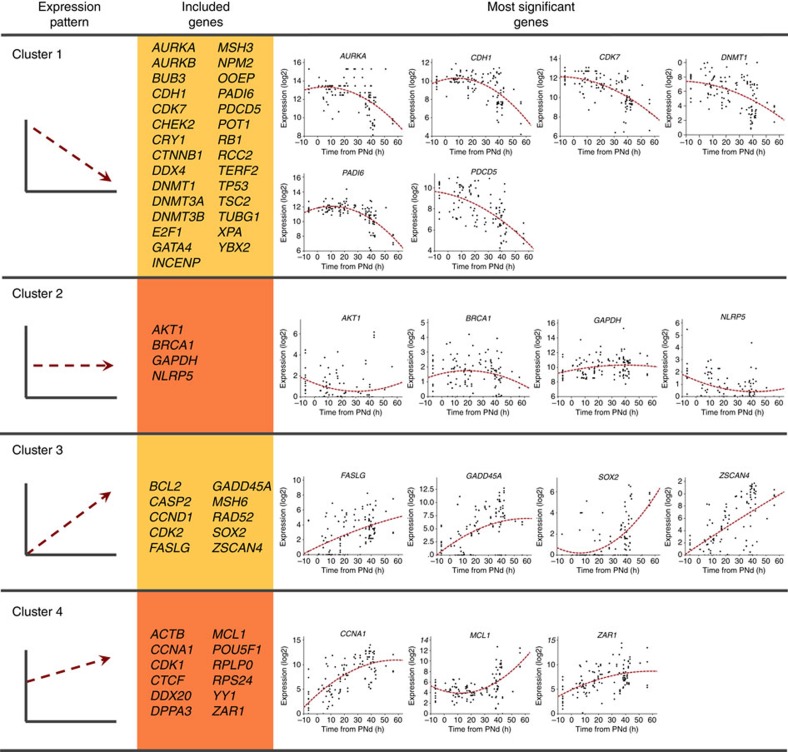
Identification of gene expression clusters during human embryo development. Significant quadratic regressions were classified into four different clusters according to the expression trend versus time. The majority of genes included in Cluster 1 (*n*=29) were expressed in the zygote stage but decreased in expression by at least twofold between the start and the final time. Cluster 2 (*n*=4) was composed of genes that showed relatively constant expression as defined by an increase or decrease in expression of less than 1 point. Cluster 3 (*n*=10) consisted of genes with an expression value lower than 2 at time zero and at least a twofold difference at the final time point. Lastly, Cluster 4 (*n*=12) comprised those genes with expression higher than 2 at time zero, and twofold or more at the last time point. The most significant regressions from each gene cluster were selected by ANOVA test with *P*<1 × 10^−6^ and fold change>10 for Clusters 1, 3, 4 and *P*<0.05 for Cluster 2. Each expression data point corresponds to the mean value obtained from three technical replicates. A baseline of *C*_t_=28 was used to obtained the showed expression values.

We then grouped the genes into four different clusters according to their expression dynamics (Fig. 3). For the interpretation of the clusters, we considered that the gene expression values for each time point were the result of inherited molecules from the gametes and/or newly synthesized molecules generated by the embryonic genome. Thus, inherited transcripts would be highly expressed at the pronuclear stage and decrease in expression as development proceeds unless they are activated by the embryonic genome. Embryonically transcribed genes, on the other hand, would increase with development and exhibit no or relatively minor transcriptional inheritance from the gametes.

Cluster 1 (*n*=29) comprised genes inherited from the gametes and showed no evidence of transcriptional activation by the embryo since expression levels decreased throughout development. On further analysis of the cluster 1 genes, we determined that the most significant annotations (*P*≤0.001, Fisher's exact test) were related to cell cycle regulation, DNA metabolism and chromosome organization (Table 2). In particular, aurora kinase A (*AURKA*), cadherin 1 (*CDH1*), cyclin-dependent kinase 7 (*CDK7*), DNA methyltransferase 1 (*DNMT1*), peptidyl arginine deiminase type VI (*PADI6*) and programmed cell death 5 (*PDCD5*) represented the major genes in this group (fold change>10 and *P*<1 × 10^−6^, ANOVA test; Fig. 3). Our findings are in accordance with previous reports^3^, wherein *PDCD5*, the cell death-related gene that inhibits the degradation of DNA damage response proteins, was present in the zygote and decreased in expression until day-3. Moreover, *AURKA*, which is involved in chromosome stabilization of the spindle, has also been shown to be highly expressed at early stages rather than initially detected at the eight-cell stage^21^.

Cluster 2 (*n*=4) was composed of genes that showed relatively constant expression and likely represent the bulk of transcripts inherited from the gametes since they were detected at the pronuclear stage, but also present at similar levels at later stages. By avoiding mRNA degradation or if degraded, compensated for by new synthesis from the embryonic genome, these genes are able to maintain stable levels throughout development. The genes that were statistically significant (*P*<0.05, ANOVA test) in this cluster were v-akt murine thymoma viral oncogene homolog 1 (*AKT1*), breast cancer 1 (*BRCA1*), glyceraldehyde-3-phosphate dehydrogenase (*GAPDH*) and NLR family, pyrin domain containing 5 (*NLRP5*; Fig. 3). As a group, these genes have known functions in monosaccharide metabolism and lipid biosynthesis as well as food and stress responses or RNA stability (Table 2). Notably, we detected high variability in *BRCA1* expression between cells from the same embryo, which could explain the discordance with previous findings since these studies detected differences between stages using the average expression of all equivalent samples^24^,^36^.

Cluster 3 (*n*=10) included genes that were activated during embryo development, but were not originally expressed in the zygote. Gene ontology analysis showed that these genes were associated with regulation of the cell cycle, particularly interphase, as well as other biological processes such as the stress response (*P*≤0.001, Fisher's exact test; Table 2). The most relevant genes in this cluster were Fas ligand (*FASLG)*, growth arrest and DNA-damage-inducible alpha (*GADD45A)*, SRY-box 2 (*SOX2)* and zinc-finger and SCAN domain containing 4 (*ZSCAN4*; fold change>10 and *P<*1 × 10^−6^, ANOVA test; Fig. 3). *FASLG* is a death receptor ligand whose expression has been shown to correlate with fragmentation in human embryos at the two- and four-cell stage^37^. We observed a gradual increase in *FASLG* expression with development starting with basal levels at the pronuclear stage to suggest that embryos do not undergo apoptosis until later in development as previously described^38^. In addition, *ZSCAN4*, which is involved in telomere maintenance, exhibited the greatest increase in expression of all the genes in this cluster (fold change=211), with considerably lower levels before PNd. This was also in accordance with previous studies showing *ZSCAN4* expression in eight-cell embryos and no expression in zygotes^30^.

Cluster 4 (*n*=12) genes also increased in expression on embryonic genome activated (EGA), but, unlike Cluster 3, were also detected at the pronuclear stage to suggest both a gametic and embryo source of transcripts. Cyclin A1 (*CCNA1)*, myeloid cell leukaemia 1 (*MCL1*) and zygote arrest 1 (*ZAR1*) showed the most significant difference in this group (fold change>10 and *P*<1 × 10^−6^, ANOVA test; Fig. 3). We detected low levels of *CCNA1* expression at the pronuclear stage with significantly increased expression on EGA as previously described^21^ and increasingly high levels of *ZAR1* beginning at the pronuclear stage. While *CCNA1* binds particular cell cycle regulators, *ZAR1* is thought to function as a maternal effect gene in mouse and human embryos^39^. However, gene ontology analysis did not show any statistically significant annotations for this cluster.

### Identification and timing of gametic versus EGA transcripts

**Figure 4:**
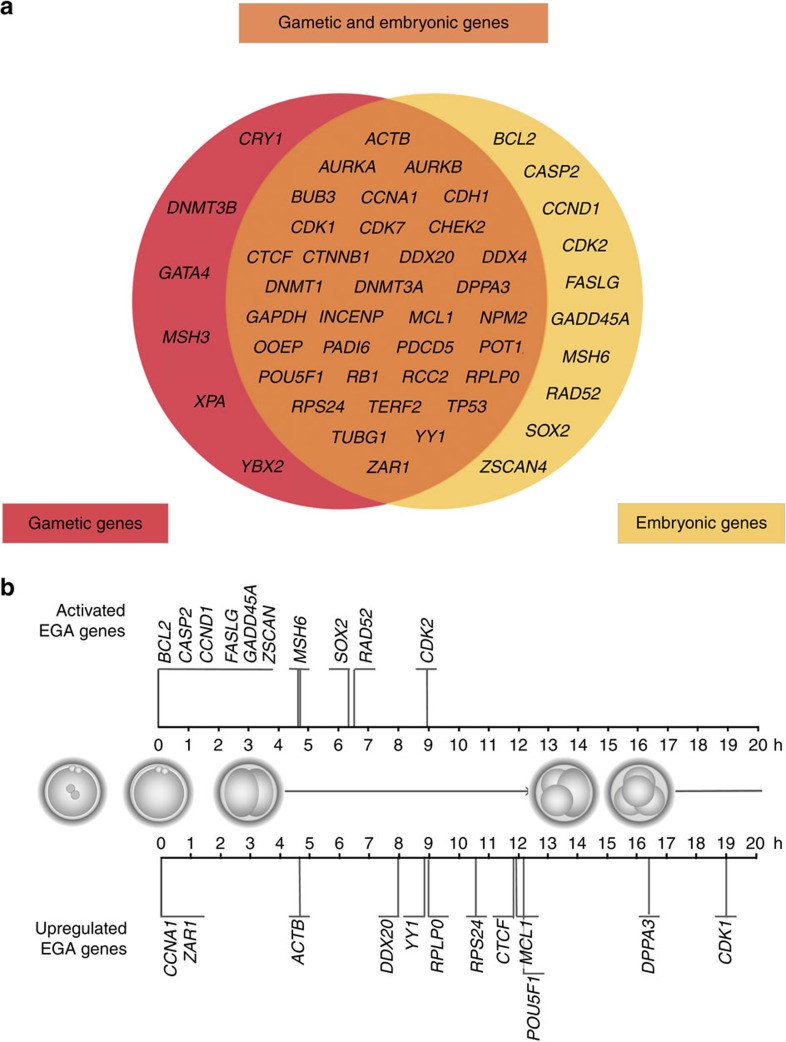
Identification and timing of gametic versus embryonic transcripts. (**a**) Gametic transcripts (*n*=40) were highly expressed at time zero, whereas EGA genes (*n*=44) were highly expressed at the final time point. The majority of these genes (*n*=34) were originally inherited from the gametes and subsequently activated by EGA. (**b**) EGA timing is shown in hours (h) after PNd. Two groups were identified according to the basal levels at the pronuclear stage: ‘Activated EGA genes' (*n*=10) that were originally absent at the zygote stage corresponded to Cluster 3 genes and ‘Upregulated EGA genes' (*n*=12) that were already present at the pronuclear stage were designated for Cluster 4 genes. A schematic representation of embryo development with normal mitotic divisions was included as a guide.

We next aimed to definitively determine which transcripts were from gametic origin or resulting from EGA. To accomplish this, we calculated the expression value at time zero for each gene using quadratic regression (Supplementary Table 2). Values higher than 2 indicated that the transcript was present in the zygote and thus provided by the gametes. When the expression values were above 2 for the final time point (Supplementary Table 2), the transcript was considered activated by EGA. From this analysis, we identified 40 genes that appeared to encode transcripts inherited from the gametes (Fig. 4a). In this group, *AURKA*, *BUB3* mitotic checkpoint protein (*BUB3*), *CDH1*, *CDK7*, developmental pluripotency-associated 3 (*DPPA3*), oocyte-expressed protein (*OOEP*) and *PADI6* were the inherited transcripts with the highest levels of expression at the zygote stage. In contrast, a total of 44 genes were clearly activated by the embryonic genome, 10 of which were undetectable in the zygote and, thus, not likely required during the earliest stages of embryo development (Fig. 4a).

For genes that showed clear activation on EGA (Clusters 3 and 4), we sought to determine the exact time in which activation occurs (Fig. 4b). The minimum of each quadratic function was calculated for this purpose and 8 of the 22 genes exhibited an increase in expression starting from PNd. Interestingly, most of the Cluster 3 genes were activated earlier in the development than Cluster 4 genes. This can be explained by the finding that they were not originally expressed in the gametes and thus needed to be activated by the EGA at the earliest stage. In contrast, we observed that the majority of the Cluster 4 genes did not need such early activation by the embryo, as there was already an initial pool of transcripts inherited from the zygote.

### Comparative gene expression analysis between ploidy groups

**Figure 5:**
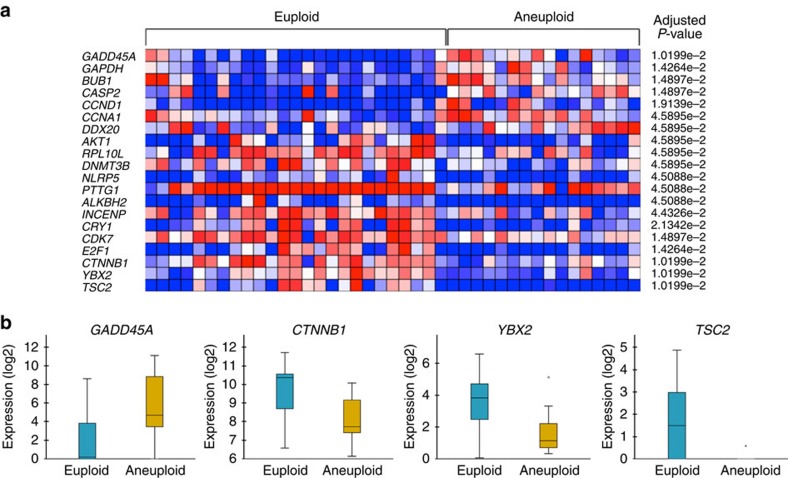
Differential gene expression in aneuploid versus euploid embryos. (**a**) Heatmap of genes showing significant differential expression (adjusted *P* value<0.05, *limma* test; *n*=20) in euploid versus aneuploid embryos during the first 30 h after PNd. Each column represents a single blastomere. Blue coloured squares show low expression, while the red colour represents high levels of gene expression with white squares indicating moderate expression (log2). Expression data correspond to the mean values obtained from three technical replicates for each gene assay. (**b**) Box plots from the most significant differentially expressed genes (adjusted *P* value=0.01, *limma* test; *n*=4) between euploid and aneuploid embryos before 30 h following PNd. A plot represents gene expression values between quartile 1 and 3, the black line inside the box is the median value and the black circles are outliers.

Ploidy and gene expression information was obtained from a total of 53 embryos with 92 cells analysed using RT–qPCR and 76 cells assessed via aCGH. To compare the expression profiles between ploidy groups without introducing bias due to differences in developmental time, two different groups were created using a cutoff of 30 h after PNd. For the first group, we obtained 41 expression profiles from 33 embryos with an incidence of aneuploidy of 39.4%; in the second group, we collected 52 expression profiles from 20 embryos with a 75.0% aneuploidy incidence. We determined that the incidence of aneuploidy was higher in the second group because of the increased frequency of mitotic errors compared with the first group. We then used Babelomics^40^ to compare gene expression levels between the two groups. In the embryos collected before 30 h, 20 of the 87 analysed genes showed statistically significant differences between euploid and aneuploid embryos (adjusted *P* value<0.05, *limma* test; Fig. 5a). We also evaluated the significant GO terms in these 20 genes to determine which molecular pathways were influenced by aneuploid generation (Supplementary Table 3). We observed that the most significant GO terms (*n*=16; *P*<0.001, Fisher's exact test) were related to cell cycle (7/16) and DNA damage (5/16). More specifically, we determined that the expression of the catenin beta 1 (*CTNNB1*), Y box-binding protein 2 (*YBX2*) and tuberous sclerosis 2 (*TSC2*) were tremendously downregulated in aneuploid embryos compared with euploid embryos (adjusted *P* value*=*0.01, *limma* test; Fig. 5b). In contrast, the DNA damage response gene, *GADD45A*, was highly expressed in aneuploid embryos and almost undetectable in euploid embryos (Fig. 5b). An analysis of gene expression 30 h after PNd, however, did not show statistically significant differences between aneuploid and euploid embryos.

### Using transcriptomic signatures to predict embryo ploidy

**Figure 6:**
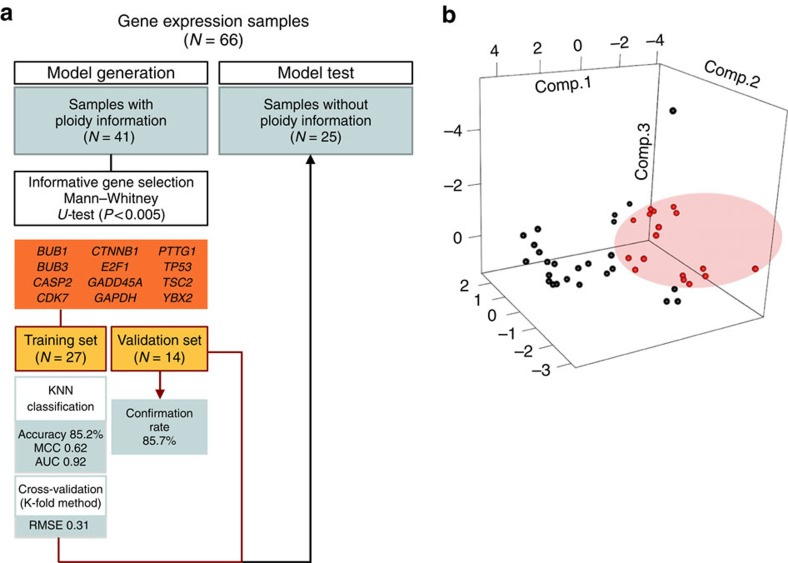
Embryo ploidy prediction model. (**a**) A diagram of each phase of the ploidy prediction model. All samples with gene expression data were used in this process. Samples with ploidy results were selected for model generation and validation. Samples without ploidy information became the prediction group. MCC, Matthews correlation coefficient; AUC, area under the curve; RMSE, root mean squared error. (**b**) Principal component analysis of cells (*n*=41) from embryos at early stages (before 30 h post PNd) on the basis of the expression of the 12 genes selected in the prediction model. Cells from euploid embryos are shown in black (*n*=25) and samples from aneuploid embryos are designated red (*n*=16).

Taking advantage of the difference in transcript expression observed between euploid and aneuploid embryos during the first 30 h of development, our next aim was to create a prediction model for embryo ploidy on the basis of a specific gene expression signature (Fig. 6a). To accomplish this, cells from which both ploidy and gene expression data were obtained with a collection time before 30 h post PNd were selected (*n*=41). Although expression values from all 87 genes were available, we focused on the most informative genes to improve the functionality of the predictor. The Mann–Whitney *U*-test was performed between the aneuploid and euploid samples to obtain 31 differentially expressed genes (*P*<0.05). Before model construction, samples were randomly split into a training group (*n*=27) and a validation group (*n*=14). Only the samples from the training group were used for the model design. To assess model accuracy, a fivefold cross-validation was performed and repeated 20 times in order to estimate the misclassification rate. From this, several models were generated depending on the number of closest neighbours evaluated for the test step and the number of genes selected for the training step. In order to obtain the most reliable predictor, we applied additional restrictive parameters and only selected genes with a *P*<0.005 (Mann–Whitney *U*-test, *n*=12). Cross-validation showed that the model with *k=*7 was the most stringent [accuracy 85.2%, Matthews correlation coefficient (MCC) 0.62, root mean squared error (RMSE) 0.31, area under the curve (AUC) 0.92]. Once selected, the predictor model was tested using the sample validation group. The confirmation rate was 85.7% (12/14) with two of the euploid samples being misclassified as aneuploid and no aneuploid samples inappropriately called as euploid. Finally, we tested the prediction model in a different group of samples collected at the same time as the training samples, but for which no ploidy results were obtained (*n*=25). The prediction model classified 11 of the samples as aneuploid and 14 as euploid with an incidence of aneuploidy of 44.0%, which is similar to the observed rate in the samples with known ploidy results at the same stage (39.0%). We also compared the time intervals between the PNd and the start of the first cytokinesis in each embryo since this was the most relevant parameter observed for assessing ploidy status. As expected, the median time was much longer in the embryos classified as aneuploid versus those predicted to be euploid (2.58 versus 1.09 h).

The predictor model identified 12 genes as applicable for the classification of euploid versus aneuploid samples and these included BUB1 mitotic checkpoint serine/threonine kinase *(BUB1)*, *BUB3*, caspase 2 (*CASP2)*, *CDK7*, *CTNNB1*, E2F transcription factor 1 (*E2F1)*, *GADD45A*, *GAPDH*, pituitary tumour-transforming 1 (*PTTG1*), *TP53*, *TSC2* and *YBX2*. With the exception of *BUB1*, *CASP2*, *GAPDH* and *GADD45A*, which were more highly expressed in aneuploid embryos, the majority of genes were upregulated in euploid embryos (Fig. 5a). Of the genes that exhibited lower expression in aneuploid embryos, many included maternally inherited genes such as *BUB3*, *CDK7*, *PTTG1*, *TSC2* and *YBX2*. Taken together, these data identify a gene subset that is differentially regulated in euploid versus aneuploid embryos and suggests that mathematical modelling on the basis of the expression of this key group of genes may provide a useful tool to largely predict the ploidy status of embryos (Fig. 6b).

## Discussion

Significant advances in single-cell genetic profiling and time-lapse imaging have dramatically increased the number of studies evaluating fundamental aspects of human preimplantation development in the past 5 years^4^,^5^,^16^,^18^,^25^. Here we report the first study to combine the analysis of complete chromosomal constitution, gene expression analysis and time-lapse culture simultaneously in the same human embryo. By evaluating all blastomeres from each embryo at a single-cell resolution across the first 3 days of development and correlating with embryo-imaging behaviour, our results provide a mathematical model predictive of ploidy status and a better understanding of early human embryogenesis.

In accordance with previous findings using either FISH^41^,^42^ or different array-based approaches^7^,^19^,^20^, we observed that at least half of the embryos in our cohort were indeed chromosomally abnormal. This further supports the notion that the high incidence of human embryonic aneuploidy at the cleavage stage is often irrespective of the fertility status, maternal age and whether from fresh versus cryopreserved cycles. Regarding the developmental kinetics of euploid versus aneuploid embryos, we found only two parameters statistically different between both groups. The most statistically significant parameter between euploid and aneuploid embryos was the time between PNd and the start of the first cytokinesis. A recent study has shown this parameter to be predictive of which embryos will reach the blastocyst stage^5^; however, no study has yet defined this parameter for assessing the ploidy status. Here we determined that the time interval between PNd and the start of the first cytokinesis was significantly longer in aneuploid than euploid embryos, suggesting that chromosome missegregation can have an impact on the length of this first mitotic cycle. Thus, our results indicate that further consideration should be given to the competency of the sperm used for fertilization as is the oocyte since several mitotic spindle components for the first cell division are paternally inherited in human embryos^43^,^44^.

Although there is some discrepancy as to whether time-lapse is actually beneficial for embryo selection^45^ and the assessment of ploidy status^46^, the first randomized control trial evaluating implantation rates, ongoing pregnancy and early pregnancy loss suggests that dynamic imaging analysis is more effective than conventional IVF techniques^9^. It remains to be determined, however, whether time-lapse imaging can also have positive impact on live birth rates, particularly in cases of single-embryo transfers. In addition, while the measurement of embryo kinetics has been shown to differentiate a large proportion of aneuploid embryos from those that are euploid, chromosomally normal and abnormal embryos that behave similarly may be indistinguishable and require genetic screening^7^,^9^,^10^,^47^. Nevertheless, it is clear from these studies that differences in findings may be because of the stage of embryonic development evaluated, whether a cell of or whole embryos were analysed, which method of ploidy assessment was used and how the imaging parameters were measured since the use of a common start point such as the time of intracytoplasmic sperm injection may have confounding effects on other overlapping parameters^2^,^46^,^48^. We note that the time between PNd and the first cytokinesis can be measured in both non-cytoplasmic sperm injection and cryopreserved embryos, in contrast to other parameters that may require the exact time of fertilization as a reference point.

Besides assessing the chromosomal status and imaging behaviour of each embryo, we also collected single-cell expression data from 87 genes during the first stages of human embryo development and showed that 55 of them exhibit a defined expression pattern. Although some of the genes have been previously described in relation to human embryo viability, most of these studies examined expression patterns in whole embryos^14^,^24^,^30^,^36^,^37^,^49^,^50^, in a pool of whole embryos^13^,^17^,^21^,^26^,^27^ and/or only at a single stage of preimplantation development^17^,^21^,^36^. In contrast, we evaluated gene expression in single blastomeres throughout multiple stages of early embryogenesis and observed a considerable amount of variability among samples. However, we addressed this complication with the use of quantile normalization, a method preferable to normalization to housekeeping genes because of individual gene variation as previously described^51^.

Since in the human embryo only a subset of genes is activated before the eight-cell stage^13^,^16^,^49^,^50^, the first hours of development rely on mRNA inherited from the gametes for survival. In this study, we identified new potential maternally or paternally derived gene products, including *CDH1*, which has been described as only expressed at the cleavage and blastocyst stage^25^. Other genes, such as *OOEP* and *PADI6*, identified as gametic in origin here have previously been reported as maternal genes in murine embryos^34^, and our data suggest that they have a conserved function in early human development. This is particularly important as recent reports suggest distinct differences in gene expression patterns between these two species^18^,^52^,^53^. In accordance with recent findings^16^, we also observed the potential activation of a subset of genes involved in cell cycle regulation, DNA metabolism or chromosome organization as early as the zygote stage. Further studies should focus on the gametic source of these early-activated genes and their precise role in human preimplantation development.

**Figure 7:**
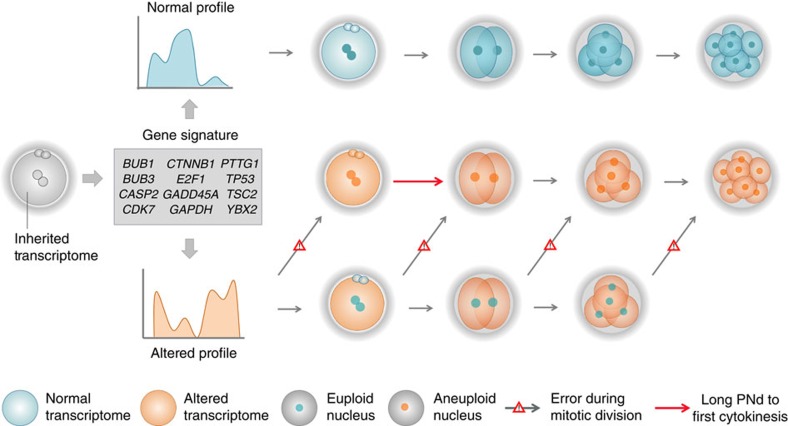
Proposed model of ploidy generation in early human embryo development. Aneuploidies in the human embryo are related to variations in both kinetics and expression profile. The inherited transcriptome at the zygote stage can be evaluated by a 12-gene signature to predict ploidy fate. When zygote transcripts levels are within normal ranges, embryo development occurs without errors in mitotic divisions and remain euploid. In contrast, when a zygote contains an altered transcriptome, mitotic errors will appear at any time throughout development. If the mitotic error happens before or during the first mitotic division, the time between PNd and the start of first cytokinesis will be longer than expected. If the mitotic error happens after the first mitotic division, on the other hand, the aneuploid embryo will not be detected by abnormal PNd to first cytokinesis kinetics but might be distinguishable by an altered transcriptome.

By combining single-cell gene expression and whole-chromosomal data analysis from the same embryo, we also identified gene expression patterns indicative of the ploidy status. Notably, differential gene expression between aneuploid and euploid embryos was observed during the first 30 h after PNd, but not later. This lack of difference may be because of potential overlap between maternal mRNA degradation and new transcription by the embryonic genome, which can occur at diverse rates between embryos of the same developmental stage and even in cells from the same embryo^15^. As uneven blastomere size is associated with a high aneuploidy incidence^54^,^55^, asymmetric distribution of transcripts caused by irregular divisions may also contribute to this finding^55^,^56^. One of the key observations of our study is the discovery of a distinct set of genes, including *CASP2*, *CCND1*, *CCNA1*, *DDX20* and *GADD45A*, that are highly expressed in aneuploid embryos. These genes further increase in expression as development proceeds, which may be explained by the mitotic propagation of chromosomal errors that occurred early in preimplantation development. We also determined that the majority of transcripts with decreased expression in aneuploid embryos belonged to Cluster 1 and, thus, likely of gametic origin. This group of genes included the cryptochrome circadian clock 1 (*CRY1*), a circadian regulator protein that has been described to have a role in female meiosis^57^, and the DNA methyltransferase 3B (*DNMT3B*). Furthermore, *YBX2*, the mouse homologue of *MSY2*, which binds maternal transcripts to avoid degradation during early embryo development^58^ and results in aberrant spindle formation when knocked out^59^, was also included in this group. Notably, both *DNMT3B* and *YBX2* were previously shown to be expressed at significantly lower levels in human embryos that had arrested at the one- or two-cell stage^3^. Taken together, our findings suggest that the inheritance of an abnormal pool of transcripts may contribute to aneuploidy in the human embryo (Fig. 7); however, it remains to be determined whether aberrant gene expression is the potential cause or consequence of chromosomal errors in the gametes during meiosis.

Finally, the most relevant finding of this study is that we were able to largely predict embryo ploidy status using a 12-gene transcriptomic signature. Although the power of the predictive model has been determined and tested with embryos from several clinics, it may be necessary to test our model on other embryo cohorts in order to extrapolate the results to other patient samples and further substantiate our findings. Moreover, while it is not currently intended for clinical use, the main goal of the prediction model is not to predict aneuploidy by itself, but to identify cellular pathways and related molecules indicative of the embryo ploidy status in culture medium or via other methods. Thus, we consider this study a keystone in the knowledge of early human embryogenesis that may lead to the development of new non-invasive diagnostic tools that can reliably predict aneuploidy generation for IVF clinical routine.

## Methods

### Experimental design

One-hundred seventeen human zygotes originating from 19 couples, with an average maternal age of 33.7±4.3 years, were thawed for this study. Eighty-five embryos survived and were cultured under time-lapse imaging (Fig. 1), obtaining a survival rate of 72.6%, which is a normal value for cryopreserved human embryos at the pronuclear stage^60^,^61^. Embryo retrieval was performed at continuous times throughout embryonic development at the pronuclear stage, and during one to seven mitotic divisions the number of the cells varied depending on the following division types: one to two, one to three or one to four cells. After embryo culture, embryos were disassembled into single blastomeres, including polar bodies from zygotes. Half of the cells of each embryo underwent whole-genome amplification (WGA) and were analysed using aCGH to determine their chromosomal status at a single-cell level. The other half was analysed using real-time RT–qPCR for 87 genes to evaluate the specific transcriptome signature in each cell. Kinetic parameters, chromosomal status and expression levels were compared and analysed for individual embryos.

### Embryo source

For this study, we obtained a large set of human embryos from previous IVF cycles after written informed consent was obtained from the Stanford University RENEW Biobank. This cohort of embryos were cryopreserved at the pronuclear stage before the assessment of quality, which have been shown to have equivalent success rates as fresh sibling zygotes^62^,^63^. It has also been recently reported that there is no effect of oocyte cryopreservation on aneuploidy incidence^64^ or kinetic parameters^65^. Embryos in the RENEW Biobank are received from several IVF clinics across the United States. De-identification was performed according to the Stanford University Institutional Review Board-approved protocol no. 10466 entitled ‘The RENEW Biobank', and molecular analysis of the embryos was in compliance with institutional regulations. No protected health information was associated with individual embryos.

### Embryo thawing and culture

Human embryos frozen at the two pronuclear stage by slow-freezing were thawed by a two-step process using the Quinn's Advantage Thaw Kit (CooperSurgical, CT, USA) as recommended by the manufacturer. The embryos were washed in Quinn's Advantage Cleavage Medium (CooperSurgical) supplemented with 10% Quinn's Advantage Serum Protein Substitute (CooperSurgical) and transferred to 100 μl drops of shared medium under mineral oil (Sigma, MO, USA). Embryos that did not survive the thaw procedure were discarded and excluded from further analysis, since this could influence the integrity of RNA and DNA within the cells, thereby affecting the results. Embryos were cultured in custom polystyrene Petri dishes (Auxogyn, CA, USA) with 12 individual microwells in the centre. Small markers (letters and numbers) were located at the edges to help with embryo identification. The dishes were prepared at least 5 h in advance and placed in the incubator to pre-equilibrate. The embryos were cultured at 37 °C with 6% CO_2_, 5% O_2_ and 89% N_2_, standard human embryo culture conditions in accordance with current clinical IVF practice.

### Time-lapse imaging

Embryos were monitored continuously using a microscope system (Auxogyn) inside a standard tri-gas incubator (Sanyo, Japan). The system consisted of an inverted digital microscope with light-emitting diode illumination, × 10 Olympus objective, automatic focus knob and 5 megapixel CMOS camera. Three types of images were taken during the culture: darkfield and brightfield images were taken automatically every 5 min and at 1 s and 500 ms of exposure time, respectively. In addition, brightfield images were also taken at 10 equidistant planes at several points throughout culture to capture images of the whole embryo. The time between multiplane captures varied depending on when the embryo was collected and one last capture was taken just before taking each embryo out of the incubator.

### Embryo disassembly and collection

The embryos were collected at different times and stages. For this purpose, the dish was taken out of the incubator for not more than 5 min to avoid affecting either the culture of the remaining embryos or the time-lapse imaging intervals. Embryos were individually transferred to 50 μl drops of Quinn's Advantage Medium with HEPES (CopperSurgical) plus 10% Human Albumin (HA; CooperSurgical) at 37 °C under mineral oil. Each procedure was performed with one single embryo at a time to maintain embryo identification and tracking. The zona pellucida was removed from each embryo by transferring the embryos to 200 μl drops of Acidified Tyrode's Solution (Millipore, MA, USA) briefly and then washing in Quinn's Advantage Medium plus 10% HA at 37 °C under mineral oil. To weaken the cellular junctions between blastomeres, embryos were incubated in 60 μl drops of Quinn's Advantage Ca^++^/Mg^++^-Free Medium with HEPES (CooperSurgical) plus 10% HA for 10 min at 37 °C under mineral oil. Embryos were disaggregated using gentle mechanical pipetting in the same medium (Supplementary Fig. 2).

Once disaggregated, counting and identification of blastomeres and polar bodies was performed. Not all blastomeres from each embryo could be harvested. Annotations referring to the cell appearance such as visible nuclei, membrane integrity and cytoplasmic anomalies were recorded during the tubing. Each sample was washed three times in 5 μl drops of PBS 1% polyvinylpyrrolidone (PVP) buffer and transferred to a sterile 0.2 ml PCR tube. All tubes were stored at −80 °C until subsequent analysis.

### Time-lapse parameter evaluation

After each experiment, brightfield images were compiled into time-lapse movies using the ImageJ software^66^. Identification labels and time stamps were included to facilitate the measurement of the imaging parameters. The imaging frames of several parameters were recorded, including PNd, each division start time, beginning of the first cytokinesis, stage of fragmentation appearance, final fragmentation percentage and the time in culture. Additional comments that could be useful for embryo evaluation such as the presence of vacuoles, abnormal cell divisions and multinucleation were also recorded. In addition, multiplane images of every embryo were assembled in a multistack file using ImageJ. Multiplane captures were used to confirm brightfield and darkfield imaging observations and to assist in the measurement of certain parameters such as PNd or percentage of fragmentation, which may be difficult to determine using just one single plane. Embryo development evaluation was completed before ploidy and gene expression analyses to ensure blinded parameter measurements.

### Detection of chromosomal abnormalities in single cells

Single-cell DNA extraction and WGA were accomplished using the Sureplex Kit (BlueGnome, Cambridge, UK) and tested using gel electrophoresis. WGA products and reference DNA (normal male and female controls) were labelled with either Cy3 or Cy5 fluorophores via the manufacturer's instructions. Control and test-labelled DNAs were combined and co-hybridized on 24sure arrays (BlueGnome) for ∼12 h. After washing, slides were scanned using Innoscan 710 (Innopsys, Carbonne, France) and the data analysed using the BlueFuse Multi software (BlueGnome). Results obtained from questionable samples, such as fragmented polar bodies or those with inconsistent aCGH profiles, were disregarded (Supplementary Fig. 3). Embryos without ploidy information were still useful to determine descriptive data about morphology, kinetics and gene expression in human embryos, as well as to train the predictor model in a blind manner.

### High-throughput single-cell gene expression analysis

Primers were designed to span exons and detect all gene isoforms whenever possible (the primers are listed in Supplementary Table 4). The procedure for gene expression analysis was adapted from the Advanced Development Protocol for Single-Cell Gene Expression Using EvaGreen DNA Binding Dye (Fluidigm, CA, USA). In brief, cDNA was prepared by adding to each individual sample the following: 9 μl RT-STA Solution (5 μl Cells Direct 2 × Reaction Mix (Invitrogen, CA, USA)); 0.2 μl SuperScript III RT Platinum Taq Mix (Invitrogen); 2.5 μl 4X Primer Mix (200 nM); and 1.3 μl DNA Suspension Buffer (Teknova, CA, USA). Reverse transcription and pre-amplification were accomplished by incubating the samples at 50 °C for 15 min and 95 °C for 2 min, followed by 18 cycles of 95 °C for 15 s and 60 °C for 4 min. Exonuclease I treatment method was used to remove unincorporated primers and the final volume was diluted twofold before qPCR. For qPCR, 2 μl of STA and Exo I-treated sample was mixed with Sample Pre-Mix solution (2.5 μl 2 × Taqman Gene Expression Master Mix (Applied Biosystems, CA, USA)); 20 × DNA Binding Dye Sample Loading Reagent (Fluidigm); 20 × EvaGreen DNA binding dye (Biotium, CA, USA). Gene assay mix solutions were prepared by adding 1.25 μl of 40 μM primer pairs with 2.5 μl 2 × Assay Loading Reagent (Fluidigm) and 1.25 μl DNA Suspension Buffer. Sample and assay mixes were loaded into 96.96 Dynamic Arrays for qPCR on a BioMark System (Fluidigm). The same technical replicates were included on each dynamic array to check for variability between arrays and ensure reliable data. Data Collection and Real-Time PCR Analysis software (Fluidigm) were used to calculate *C*_t_ values from the melt curve of each gene assay.

### Gene expression data-processing

Raw data were normalized in order to avoid variability between chips and allow comparison between blastomeres from different developmental stages. As gene activation during embryo development may not be simultaneous in embryos of similar stage or between blastomeres within the same embryo, normalization using housekeeping genes was not performed. Instead, a quantile normalization method was applied using *limma* package^67^ for R^68^. This method adjusts the overall expression levels by making the distribution for all samples equal, thereby allowing us to compare expression values between cells from different stages, or cells from the same embryo with different sizes. An assumed baseline *C*_t_ value of 28 or below was included on the basis of previous findings^69^ and all *C*_t_ values higher than this value were called as no expression. Similarly, all samples with questionable results such as a disproportionately high number of failed gene assays or unusual melt curves were discarded (Supplementary Fig. 3). Final expression values were obtained by subtracting *C*_t_ values from the *C*_t_ baseline value of 28.

### Statistical analysis of gene expression

Data analysis such as outliers, statistical tests and regression models were performed using IBM SPSS Statistics Version 21. Saphiro–Wilk test was first performed to check normal distribution of variables. If a normal distribution, *t*-test (for two groups) or one-way ANOVA (for more than two groups) was performed. When not normally distributed, the non-parametric Mann–Whitney *U*-test was performed to assess differences between groups. For gene expression models we performed quadratic regression and ANOVA test was performed to evaluate the regression accuracy. Babelomics was the selected platform for the analysis of the gene expression data^40^. The functional analysis tool, FatiGO, was used to detect over-represented functional annotations in a cluster of genes and the class comparison tool assisted in the detection of genes differentially expressed between groups. Comparative expression between aneuploid and euploid embryos from the same time point was performed in the class comparison tool using the *limma* test in Babelomics^40^ and Benjamini and Hochberg test was selected to estimate the false discovery rate.

### Ploidy predictor model

The prediction model was built using the *k*-nearest neighbours algorithm (k-NN) in the Babelomics platform^40^, which consists of a function for the measurement of distances between samples on the basis of gene expression profiles. To avoid bias during model generation, sample-split was performed. Two-thirds of the samples were randomly selected and grouped as a training set and the other one-third of the samples became the validation set, which was used to test the model once generated. Each sample from the training group was assigned a class: euploid or aneuploid. For a test sample, the model was assigned a class attending to the most represented among the closest *k* samples. Several models were generated on the basis of the number of neighbours that were evaluated for the prediction. In order to select the most accurate model, a *k*-fold cross-validation was performed. In this method, the data set was automatically split into *k* partitions and *k–1* was used for model training and error estimation, respectively. This process was complete when all samples were tested and repeated several times in order to improve prediction accuracy.

## Additional information

**How to cite this article:** Vera-Rodriguez, M. *et al*. Prediction model for aneuploidy in early human embryo development revealed by single-cell analysis. *Nat. Commun.* 6:7601 doi: 10.1038/ncomms8601 (2015).

## Supplementary Material

Supplementary Figures and Supplementary Tables Supplementary Figures 1-3 and Supplementary Tables 1-4

Supplementary Movie 1 Time-lapse imaging showing an abnormal first division (1to3). On the left, an embryo with normal mitotic divisions. On the right, an embryo with an abnormal first division from 1 to 3 cells. Multi-capture images from the last frame have been included at the end of the movie.

Supplementary Movie 2 Time-lapse imaging showing an abnormal first division (1to4). On the left, an embryo with normal mitotic divisions. On the right, an embryo with an abnormal first division from 1 to 4 cells. Multi-capture images from the last frame have been included at the end of the movie.

Supplementary Movie 3 Time-lapse imaging showing an abnormal third division (1to3). On the left, an embryo with normal mitotic divisions. On the right, an embryo with an abnormal third division from 1 to 3 cells. Multi-capture images from the last frame have been included at the end of the movie.

## Figures and Tables

**Table 1:** Kinetic parameters in euploid versus aneuploid embryos.

	All embryos			Normal divisions			Abnormal divisions		
	N	Median	IQR	N	Median	IQR	N	Median	IQR
*PNd to first cytokinesis (h)*									
** **Euploid	22	2.4*	(2.1; 2.9)	18	2.3^‡^	(2.1; 2.9)	4	2.9	(0.9; 3.1)
** **Aneuploid	26	2.8*	(2.5; 3.3)	14	2.8^‡^	(2.5; 3.3)	12	2.9	(2.5; 7.9)
									
*First cytokinesis (min)*									
** **Euploid	22	15.0	(13.8; 26.3)	18	15.0	(10.0; 21.3)	4	25.0	(16.3; 60.0)
** **Aneuploid	26	20.0	(13.8; 31.3)	14	20.0	(10.0; 31.3)	12	22.5	(15.0; 37.5)
									
*Two to three cells (h)*									
** **Euploid	14	11.3	(1.4; 12.2)	10	11.7	(11.1; 12.6)	4	1.3	(0.5; 4.3)
** **Aneuploid	23	11.4	(0.8; 12.6)	11	12.5	(11.8; 12.9)	12	0.8	(0.1; 2.4)
									
*Three to four cells (h)*									
** **Euploid	12	0.8^†^	(0.2; 1.3)	10	0.8	(0.4; 1.3)	2	5.4	(0.1; NA)
** **Aneuploid	20	2.4^†^	(0.9; 8.4)	11	1.7	(0.8; 2.7)	9	4.4	(1.6; 12.3)
									
*Four to five cells (h)*									
** **Euploid	5	5.3	(1; 13.8)	4	8.8	(2.9; 14.6)	1	0	(0; 11.2)
** **Aneuploid	25	9.9	(0.8; 12.3)	6	12.8	(11.7; 15.7)	9	2.7	(0.3; 9)
									
*Five to six cells (h)*									
** **Euploid	5	7.2	(1.4; 9.3)	4	4.9	(0.7; 7.4)	1	11.2	(11.2; 4.3)
** **Aneuploid	12	1.6	(0.4; 3.8)	6	1.6	(0.5; 2.7)	6	1.6	(0.3; 13.8)
									
*Six to seven cells (h)*									
** **Euploid	4	1	(0.6; 3.5)	3	0.8	(0.5; NA)	1	4.3	(4.3; 0.8)
** **Aneuploid	12	2	(1; 8.5)	6	1.5	(0.5; 3.9)	6	5.1	(1.2; 13.2)
									
*Seven to eight cells (h)*									
** **Euploid	4	0.8	(0.6; 1.1)	3	0.8	(0.5; NA)	1	0.8	(0.8; 0.5)
** **Aneuploid	10	0.7	(0.4; 3.4)	6	1.5	(0.4; 3.4)	4	0.6	(0.3; 4.9)
									
*Eight to nine cells (h)*									
** **Euploid	1	0.5	(0.5; 0.5)	0	NA	(NA; NA)	1	0.5	(0.5; NA)
** **Aneuploid	2	2.5	(1.6; NA)	0	NA	(NA; NA)	2	2.5	(1.6; NA)

IQR, interquartile range (Q1; Q3); NA, not applicable; PNd, pronuclei disappearance.

Kinetic parameters were calculated for every aneuploid and euploid embryo (‘All embryos'). In addition, they were classified according to the type of divisions (‘Normal divisions' and ‘Abnormal divisions'), since one abnormal division may alter all subsequent kinetic parameters, which are calculated based on the cell stage of the embryo. *,†,‡*P*<0.05 (Mann–Whitney *U*-test).

**Table 2:** Gene ontology classifications for each cluster.

GO:Term	Term name	C	G	Adjusted *P* value
*Cluster 1*				
GO:0007049	Cell cycle	15	937	1.09E−10
GO:0006259	DNA metabolic process	12	635	6.03E−09
GO:0022402	Cell cycle process	11	638	1.00E−07
GO:0009892	Negative regulation of metabolic process	11	707	2.20E−07
GO:0009890	Negative regulation of biosynthetic process	10	561	3.68E−07
GO:0051276	Chromosome organization	10	627	8.79E−07
GO:0009893	Positive regulation of metabolic process	11	858	9.03E−07
GO:0051726	Regulation of cell cycle	8	313	9.03E−07
GO:0000278	Mitotic cell cycle	9	469	9.03E−07
GO:0000279	M phase	8	386	3.88E−06
GO:0009790	Embryonic development	9	608	6.72E−06
GO:0033044	Regulation of chromosome organization	4	28	1.18E−05
GO:0001701	*In utero* embryonic development	6	183	1.67E−05
GO:0043009	Chordate embryonic development	7	344	2.63E−05
GO:0051053	Negative regulation of DNA metabolic process	4	37	2.63E−05
GO:0009792	Embryonic development ending in birth or egg hatching	7	348	2.64E−05
GO:0010628	Positive regulation of gene expression	8	578	4.53E−05
GO:0034984	Cellular response to DNA damage stimulus	7	382	4.53E−05
GO:0050790	Regulation of catalytic activity	9	851	6.49E−05
GO:0006974	Response to DNA damage stimulus	7	422	7.55E−05
GO:0006366	Transcription from RNA polymerase II promoter	9	882	7.90E−05
GO:0000087	M phase of mitotic cell cycle	6	269	8.95E−05
GO:0016481	Negative regulation of transcription	7	450	1.00E−04
GO:0016568	Chromatin modification	6	282	1.07E−04
GO:0006461	Protein complex assembly	8	682	1.12E−04
GO:0031328	Positive regulation of cellular biosynthetic process	8	700	1.25E−04
GO:0006275	Regulation of DNA replication	4	65	1.25E−04
GO:0010629	Negative regulation of gene expression	7	491	1.46E−04
GO:0000075	Cell cycle checkpoint	4	77	2.16E−04
GO:0008156	Negative regulation of DNA replication	3	26	4.36E−04
GO:0051259	Protein oligomerization	5	217	4.91E−04
GO:0051716	Cellular response to stimulus	8	865	4.99E−04
GO:0033554	Cellular response to stress	7	623	5.80E−04
GO:0032259	Methylation	4	106	6.07E−04
GO:0006260	DNA replication	5	232	6.07E−04
GO:0006306	DNA methylation	3	33	7.06E−04
GO:0051096	Positive regulation of helicase activity	2	3	8.39E−04
GO:0032206	Positive regulation of telomere maintenance	2	3	8.39E−04
GO:0030521	Androgen receptor signalling pathway	3	37	8.96E−04
GO:0007067	Mitosis	5	260	8.96E−04
GO:0051128	Regulation of cellular component organization	6	458	9.51E−04
GO:0043086	Negative regulation of catalytic activity	5	266	9.51E−04
				
*Cluster 2*				
GO:0006006	Glucose metabolic process	3	163	6.65E−04
GO:0010907	Positive regulation of glucose metabolic process	2	12	6.65E−04
GO:0019318	Hexose metabolic process	3	201	6.65E−04
GO:0032094	Response to food	2	15	6.65E−04
GO:0032369	Negative regulation of lipid transport	2	13	6.65E−04
GO:0032770	Positive regulation of monooxygenase activity	2	17	6.65E−04
GO:0034405	Response to fluid shear stress	2	8	6.65E−04
GO:0045598	Regulation of fat cell differentiation	2	13	6.65E−04
GO:0048009	Insulin-like growth factor receptor signalling pathway	2	16	6.65E−04
GO:0050995	Negative regulation of lipid catabolic process	2	17	6.65E−04
GO:0050999	Regulation of nitric-oxide synthase activity	2	13	6.65E−04
GO:0051000	Positive regulation of nitric-oxide synthase activity	2	7	6.65E−04
GO:0043487	Regulation of RNA stability	2	14	6.65E−04
GO:0005996	Monosaccharide metabolic process	3	236	7.07E−04
GO:0008633	Activation of pro-apoptotic gene products	2	19	7.08E−04
GO:0043029	T-cell homeostasis	2	20	7.30E−04
GO:0015909	Long-chain fatty acid transport	2	22	8.21E−04
GO:0043491	Protein kinase B signalling cascade	2	23	8.43E−04
GO:0046889	Positive regulation of lipid biosynthetic process	2	25	9.34E−04
GO:0002260	Lymphocyte homeostasis	2	28	1.00E−03
GO:0045862	Positive regulation of proteolysis	2	28	1.00E−03
GO:0051353	Positive regulation of oxidoreductase activity	2	28	1.00E−03
				
*Cluster 3*				
GO:0022402	Cell cycle process	6	643	9.19E−05
GO:0051325	Interphase	4	115	9.19E−05
GO:0051329	Interphase of mitotic cell cycle	4	109	9.19E−05
GO:0007049	Cell cycle	6	948	4.28E−04
GO:0009411	Response to ultraviolet	3	55	7.31E−04
GO:0033554	Cellular response to stress	5	625	9.57E−04
GO:0033273	Response to vitamin	3	69	9.57E−04
GO:0046661	Male sex differentiation	3	71	9.57E−04

C, number of genes annotated by the given term in the test set; G, number of genes annotated by the given term in the reference set; GO, gene ontology.

Two-tailed Fisher's exact test was performed to detect most significant GO terms. Only results with *P*≤0.001 are shown. GO terms with *P*≤0.001 were not found for Cluster 4 genes.
