# Supplementary material for: Prediction model for aneuploidy in early human embryo development revealed by single-cell analysis
Source: Nat Commun. 2015 Jul 7;6:7601. doi: 10.1038/ncomms8601 (PMC4506544; doi:10.1038/ncomms8601)
Supplement: Supplementary Figures and Supplementary Tables — Supplementary Figures 1-3 and Supplementary Tables 1-4 [file ncomms8601-s1.pdf]

Supplementary information

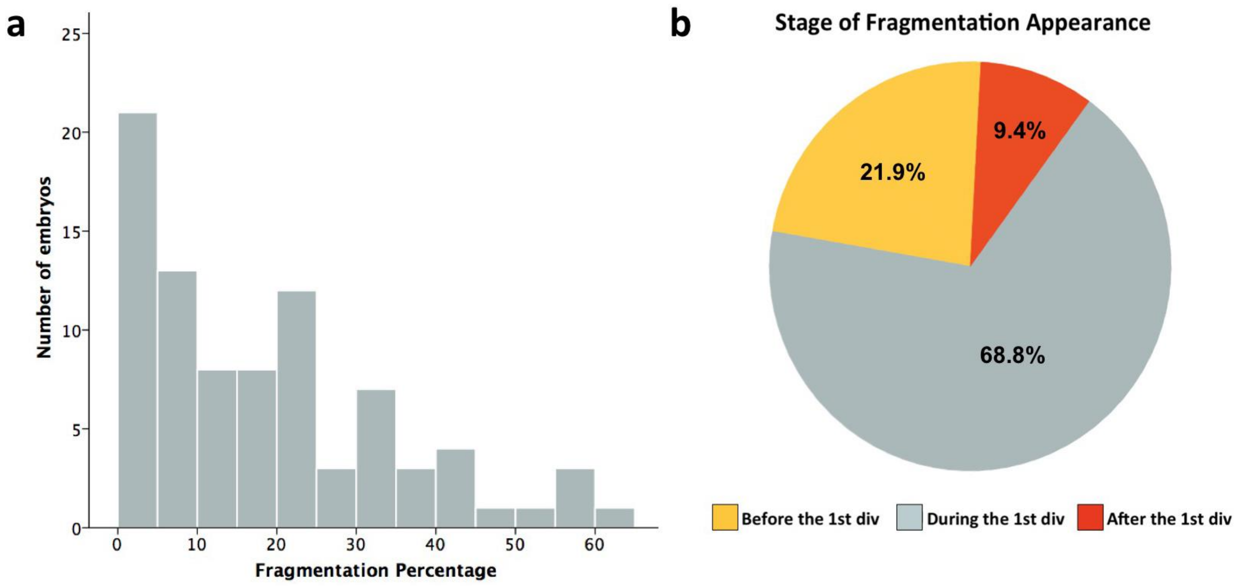

Supplementary Figure 1. Cellular fragmentation dynamics in human embryos.
(**a**) The distribution of embryos according to the percentage of fragmentation at collection (n=85). The fragmentation percentage was confirmed via three-dimensional multi-plane imaging. (**b**) A pie graph showing that almost all fragmentation appears before (n=14) or during the first division (n=44) with a much smaller percentage appearing after that (n=6). Note that each embryo was annotated by the stage in which fragmentation appeared for the first time.

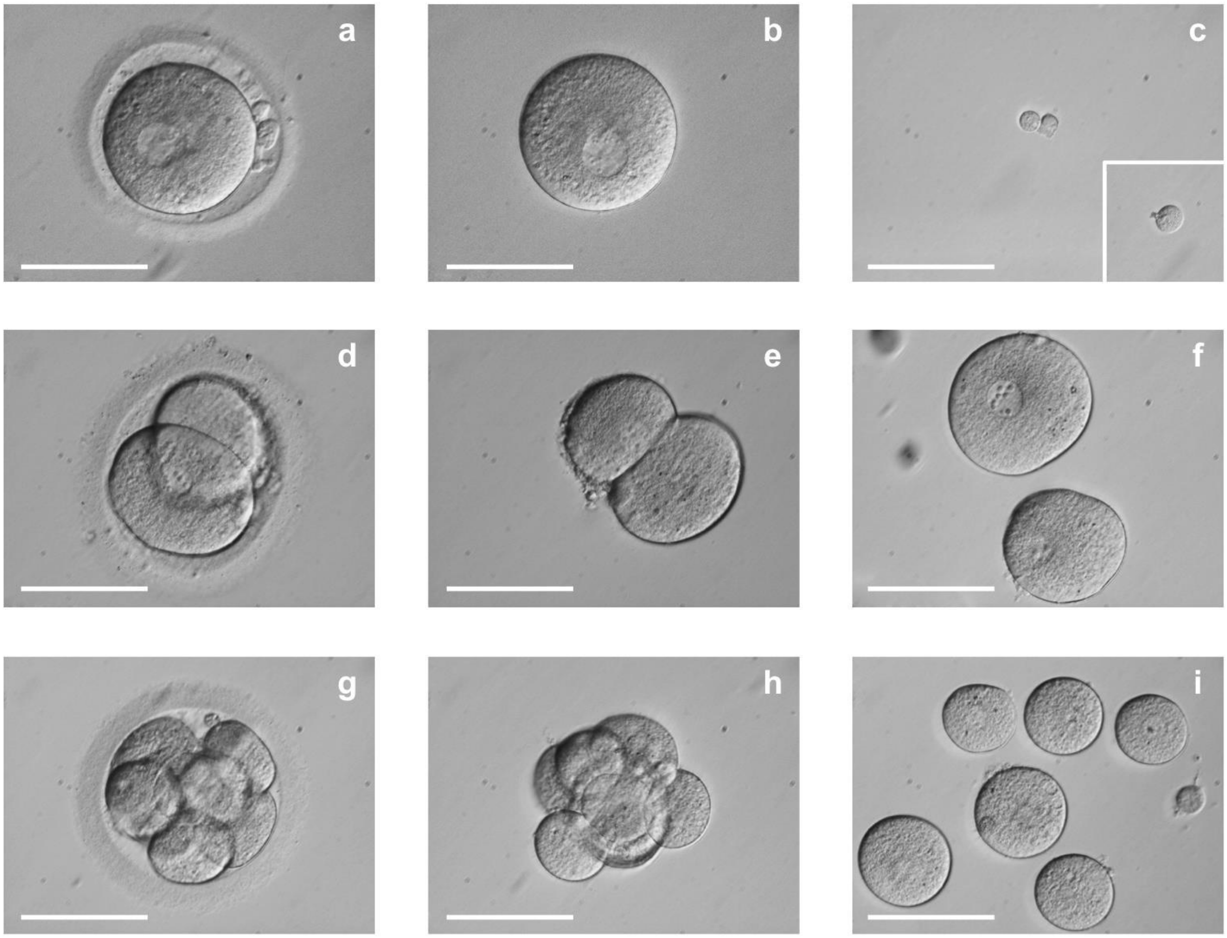

Supplementary Figure 2. Representative images of embryo disassembly at different stages. Embryos were removed and disaggregated at different times during development. An embryo at the 1-cell stage (**a**) was detached from its zona pellucida and separated into zygote (**b**) and polar bodies (**c**). Note that the differentiation between first and second polar bodies was not possible. Another embryo at the 2-cell stage (**d**) had its zona pellucida removed (**e**) and was disassembled into individual blastomeres (**f**). An embryo at the 8-cell stage (**g**) was released from the zona pellucida (**h**) and disaggregated into individual cells (**i**). Note that in fragmented embryos, fragments were separated from blastomeres by mechanical pipetting since they may potentially interfere in the results. Scale bar, 100 μm.

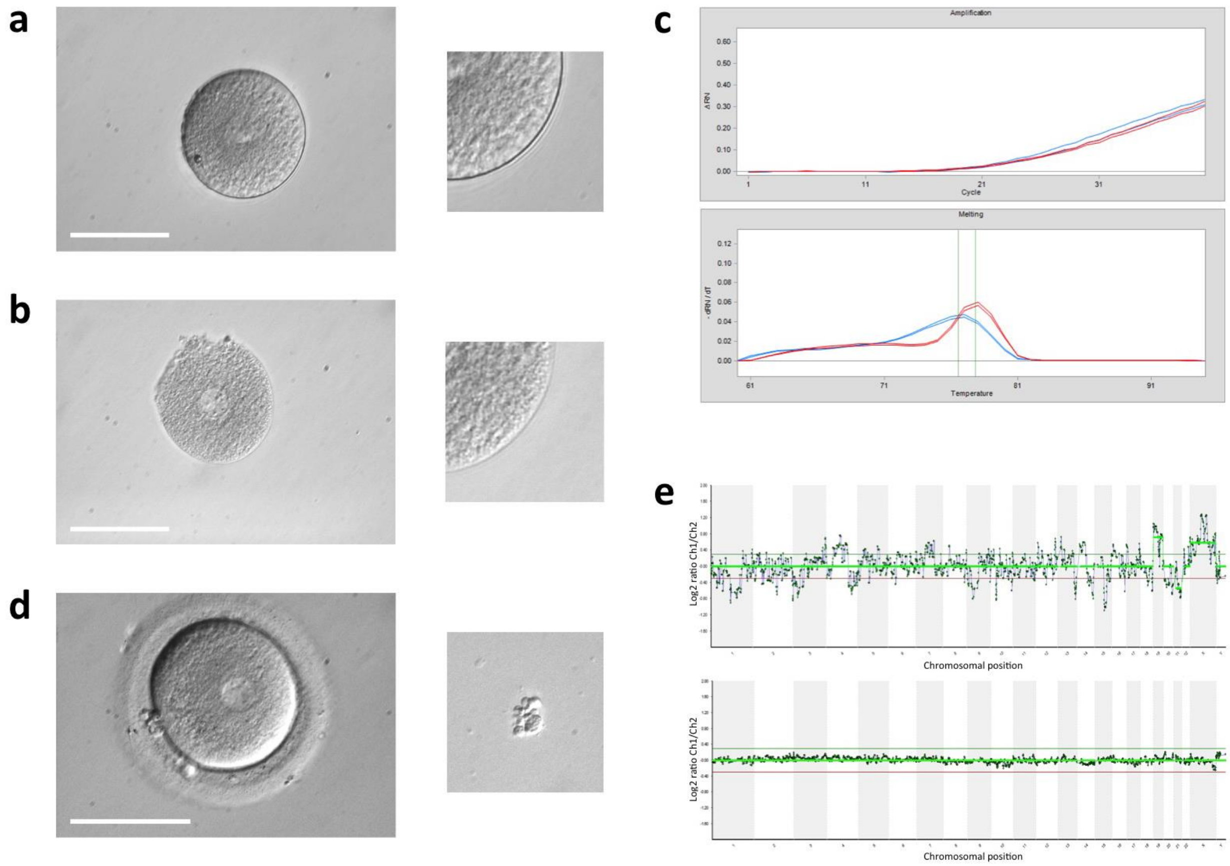

Supplementary Figure 3. Examples of exclusion criteria.

Since the integrity of the cell membrane could alter gene expression results, only blastomeres with an intact membrane were included (**a**), whereas blastomeres with a compromised membrane (**b**) were disregarded in the analysis. This is based on qPCR curves from blastomeres with an intact membrane (**c**, red lines) and a compromised membrane (**c**, blue lines). Ploidy results were also discarded in cases of a zygote with polar body fragmentation (**d**) since DNA from fragmented polar bodies may be degraded and show an aCGH profile with amplification failure (**e,** top) rather than the typical aCGH profile (**e**, bottom). Scale bar, 100 μm.

Supplementary Table 1. **Developmental kinetics of all cultured embryos.**

Parameter	N	Q1 (h)	Median (h)	Q3 (h)
PNd to First cytokinesis	48	2.33	2.71	3.15
First cytokinesis	67	0.25	0.33	0.50
2 to 3-cell	47	0.92	11.42	14.50
3 to 4-cell	39	0.58	1.25	4.42
4 to 5-cell	21	0.71	8.08	12.25
5 to 6-cell	17	0.46	2.25	7.29
6 to 7-cell	16	0.79	1.54	6.44
7 to 8-cell	14	0.50	0.79	2.17
8 to 9-cell	3	0.50	1.58	NA

All parameters with the number (N) of embryos analyzed and data distribution times (Q1, 25th percentile; Median, 50th percentile; Q3, 75th percentile) shown in hours (h). PNd, pronuclei disappearance; first cytokinesis, time between the appearance of a cleavage furrow and completion of daughter cells separation.

Supplementary Table 2. Quadratic regression analysis of gene expression profiles.

GENE	EQUATION COEFFICIENTS				PREDICTED EXPRESSION VALUES	
	a	*b(x)*	*c(x^2)*	*P*-value	At start time	At final time
ACTB	5.91	-0.01	6.48E-04	3.2E-04	5.91	7.60
AKT1	1.32	-0.05	8.01E-04	2.6E-02	1.32	1.05
AURKA	13.30	0.01	-1.37E-03	<1.0E-06	13.30	9.81
AURKB	9.71	-1.2E-03	-3.86E-04	4.8E-03	9.71	8.43
BCL2	0.87	0.02	8.54E-04	1.1E-04	0.87	4.50
BRCA1	1.56	0.02	-5.83E-04	4.6E-02	1.56	0.92
BUB3	10.63	-0.03	-1.69E-04	2.6E-03	10.63	8.42
CASP2	0.98	0.05	-6.55E-04	8.3E-04	0.98	2.00
CCNA1	3.92	0.24	-2.06E-03	<1.0E-06	3.92	10.93
CCND1	0.64	0.03	7.00E-04	2.0E-06	0.64	4.36
CDH1	10.17	0.03	-1.68E-03	<1.0E-06	10.17	6.50
CDK1	6.95	-0.10	2.63E-03	1.0E-02	6.95	9.60
CDK2	1.74	-0.01	6.24E-04	5.8E-03	1.74	3.07
CDK7	12.05	-0.02	-8.09E-04	<1.0E-06	12.05	8.51
CHEK2	6.95	-0.01	-6.11E-04	1.2E-05	6.95	4.28
CRY1	2.85	-0.10	9.54E-04	<1.0E-06	2.85	0.09
CTCF	3.83	-0.03	1.34E-03	4.3E-04	3.83	6.24
CTNNB1	9.19	-0.02	-2.33E-04	4.7E-04	9.19	7.28
DDX20	2.99	-0.01	9.04E-04	2.8E-02	2.99	5.02
DDX4	4.46	-0.02	-1.54E-04	8.0E-06	4.46	2.66
DNMT1	7.22	-0.03	-8.63E-04	<1.0E-06	7.22	3.03
DNMT3A	7.49	0.03	-1.17E-03	8.2E-05	7.49	5.54
DNMT3B	4.23	-1.6E-03	-8.87E-04	1.2E-05	4.23	1.36
DPPA3	11.85	-0.05	1.67E-03	1.7E-02	11.85	14.02
E2F1	1.25	-0.05	5.09E-04	<1.0E-06	1.25	0.23
FASLG	1.03	0.09	-3.23E-04	<1.0E-06	1.03	4.82
GADD45A	1.87	0.17	-1.42E-03	<1.0E-06	1.87	6.94
GAPDH	9.59	0.04	-4.36E-04	3.6E-02	9.59	10.19
GATA4	3.03	0.01	-9.43E-04	8.0E-06	3.03	0.36
INCENP	5.59	-0.01	-3.15E-04	3.6E-04	5.59	4.06
MCL1	4.32	-0.07	2.89E-03	<1.0E-06	4.32	9.55
MSH3	4.26	0.02	-1.16E-03	2.0E-06	4.26	1.65
MSH6	0.07	-0.01	1.01E-03	<1.0E-06	0.07	2.72

NLRP5	1.32	-0.04	5.22E-04	7.5E-05	1.32	0.51
NPM2	7.27	0.04	-1.65E-03	6.0E-06	7.27	4.54
OOEP	11.01	-0.03	-2.27E-04	2.4E-05	11.01	8.45
PADI6	11.83	0.04	-2.02E-03	<1.0E-06	11.83	7.89
PDCD5	9.41	-0.03	-7.92E-04	<1.0E-06	9.41	5.18
POT1	8.52	-0.02	-1.57E-04	1.8E-02	8.52	6.84
POU5F1	8.10	-0.02	9.06E-04	8.8E-03	8.10	9.70
RAD52	0.23	-0.01	1.09E-03	2.0E-06	0.23	2.85
RB1	6.20	-0.02	-6.55E-04	5.2E-03	6.20	2.99
RCC2	8.81	-0.01	-3.12E-04	4.6E-05	8.81	7.34
RPLP0	6.76	-0.03	1.49E-03	7.0E-06	6.76	9.96
RPS24	5.95	-0.02	1.09E-03	9.9E-03	5.95	8.08
SOX2	0.28	-0.02	1.88E-03	<1.0E-06	0.28	4.82
TERF2	7.16	-0.01	-2.11E-04	2.7E-05	7.16	5.81
TP53	5.64	0.01	-1.11E-03	1.0E-06	5.64	2.48
TSC2	1.33	-0.01	-1.60E-04	2.9E-02	1.33	0.17
TUBG1	6.11	-0.03	7.91E-05	1.2E-04	6.11	4.79
XPA	2.89	-0.06	2.92E-04	<1.0E-06	2.89	0.18
YBX2	3.38	-0.05	1.56E-05	<1.0E-06	3.38	0.59
YY1	7.43	-0.03	1.85E-03	2.0E-06	7.43	11.41
ZAR1	4.88	0.13	-1.08E-03	<1.0E-06	4.88	8.66
ZSCAN4	1.61	0.15	-1.37E-04	<1.0E-06	1.61	9.33

ANOVA test was performed to evaluate the regression accuracy. Only genes with P-value<0.05 are shown. Predicted log2 values (y) were obtained by using the quadratic equation ($y=a+bx+cx^2$) for the start time (x = 0) and the final time (x = 56h).

Supplementary Table 3. Significant Gene Ontology terms for genes differentially expressed between aneuploid and euploid embryos.

GO:Term	Term name	C	G	Adjusted *P*-value
GO:0007049	Cell cycle	11	941	1.14E-07
GO:0022402	Cell cycle process	9	640	1.19E-06
GO:0000278	Mitotic cell cycle	8	470	1.98E-06
GO:0051726	Regulation of cell cycle	7	314	2.94E-06
GO:0051301	Cell division	6	281	4.51E-05
GO:0051716	Cellular response to stimulus	8	865	1.05E-04
GO:0006259	DNA metabolic process	7	640	1.56E-04
GO:0033554	Cellular response to stress	7	623	1.56E-04
GO:0034984	Cellular response to DNA damage stimulus	6	383	1.56E-04
GO:0006974	Response to DNA damage stimulus	6	423	2.38E-04
GO:0051329	Interphase of mitotic cell cycle	4	109	4.29E-04
GO:0019953	Sexual reproduction	6	484	4.29E-04
GO:0051325	Interphase	4	115	4.52E-04
GO:0051246	Regulation of protein metabolic process	6	508	4.52E-04
GO:0048609	Reproductive process in a multicellular organism	6	506	4.52E-04
GO:0006281	DNA repair	5	330	9.96E-04

Only GO terms with adjusted *P*-value<0.001 (Fisher exact test) are shown. C, Number of genes annotated by the given term in the test set; G, Number of genes annotated by the given term in the reference set.

Supplementary Table 4. Primers for gene expression analysis by RT-qPCR.

Target Gene	Gene ID	Forward primer	Reverse primer	Product Length
AHR	196	AGCCGGTGCAGAAAACAGTA	GGTCTCTATGCCGCTTGGAA	75
AKT1	207	CCATCACACCACCTGACCAA	CGAGTAGGAGAACTGGGGGA	83
ALKBH2	121642	CTGTTTGGCAAAGCTGAGGC	GGGGCCAGTTCTCTTTCATCA	127
APC	324	GGGCTTACTAATGACCACTACA	TGCAGCCTTTCATAGAGCATA	118
ATM	472	TGCTTGCTGTTGTGGACTA	ATCCAGCCAGAAAGCATCA	80
AURKA	6790	GGTGGTCAGTACATGCTCCA	GCATCCGACCTTCAATCATTTCA	90
AURKB	9212	ATGGAGAATAGCAGTGGGACAC	CAGAGGACGCCCAATCTCAA	78
BAD	572	CTCCGGAGGATGAGTGACGA	CACCAGGACTGGAAGACTCG	125
BAX	581	GGGTTGTCGCCCTTTTCTAC	TCTTGGATCCAGCCCAACA	136
BCL2	596	AGGGTACGATAACCGGGAGAT	CATCCCACTCGTAGCCCCTC	74
BRCA1	672	AAGACTGCTCAGGGCTATCC	CCATTTCCTGCTGGAGCTTTA	96
BRCA2	675	ATGCAGCAGACCCAGCTTA	TCCTTTTGTTCAGCAGATTCCA	148
BUB1	699	ACAAGCTTCCAGTGGAGTCA	AATCCAAAGTCGCCTGGGTA	84
BUB3	9184	CTGCATACGAGCGTTTCCAAA	GGCTTGGGTCCAAATACTCAAC	86
CASP2	835	AACTGCCCAAGCCTACAGAA	TTGGTCAACCCCACGATCA	84
CCNA1	8900	GCTCGTAGGAACAGCAGCTA	CAAACTCGTCTACTTCAGGAGGATA	74
CCND1	595	ATCTACACCGACAACTCCATCC	GGTTCCACTTGAGCTTGTTCAC	79
CCNE1	898	ATACTTGCTGCTTCGGCCTT	TCAGTTTTGAGCTCCCCGTC	148
CCT3	7203	CCCGATGTGGTCATCACTGAA	CTCTGCGGATGGCTGTGATA	85
CDH1	999	CGTCACCACAAATCCAGTGAAC	TACTGCTGCTTGGCCTCAAA	78
CDK1	983	CCTAGTACTGCAATTCGGGAAA	CCTGCATAAGCACATCCTGAA	85
CDK2	1017	TGGGCCCGGCAAGATTTTAG	TGTTAGGGTCGTAGTGCAGC	91
CDK7	1022	GGGAGCCCCAATAGAGCTTATA	CTACACCATACATCCTAGCTCCA	91
CETN2	1069	GGAGTTGGGTGAGAACCTGAC	TCTTGCTCACTGACCTCTCC	90
CFL1	1072	GCTCCAAGGACGCCATCA	TCCTTGACCTCCTCGTAGCA	79
CHEK1	1111	TGGTACAACAAACCCCTCAA	CACTGGGAGACTCTGACAC	76
CHEK2	11200	GCCCTTCAGGATGGATTTGC	ACAGCTTTTGTCCCTCCCAAA	72
CLOCK	9575	CCTGAGACAGCTGCTGACAA	ACGGCCGTGTGAGATGATTT	149
CRY1	1407	AACCAGCAGATGTGTTTCCC	CCTTTCCAAAGGGCTCAGAA	87
CTCF	10664	GCGGCTTTTGTCTGTTCTAA	CTGGGCCAGCACAATTATCA	85
DDX20	11218	ATGCATCGGATTGGGAGAGC	TTTTCTTCCTCTCCCCGGCA	80
DIAPH1	1729	TCCCTTCGTGTGTCTCTCAA	TAAGGAGGCCAAGCCTTCA	75
DNMT1	1786	GCCATTGGCTTGGAGATCA	AGCAGCTTCCTCCTCCTTTA	84
DNMT3A	1788	AGCCTCAATGTTACCCTGGAAC	TACGCACACTCCAGAAAGCA	83

DNMT3B	1789	GTGAAGCACGAGGGGAATATCA	TTCCGCCAATCACCAAGTCA	100
DPPA3	359787	AAGACCAACAAACAAGGAGCC	TCCCATCCATTAGACACGCAG	90
E2F1	1869	AGCTCATTGCCAAGAAGTCCAA	TCCTGGGTCAACCCCTCAA	94
ECT2	1894	GCTGTGTCGACATGTAGCTAA	CAAAGGATTCTGGATCAGCAGTA	80
FASLG	356	CTTGGTAGGATTGGGCCTGG	TGTGTGCATCTGGCTGGTAG	91
GADD45A	1647	GCGACCTGCAGTTTGCAATA	CTTTCGGTCTTCTGCTCTCCA	63
INCENP	3619	AGCAGAAGGCTTGCCAAGAA	ATTCAGGAGCCTCTCCAGGTAA	87
MAD2L1	4085	AGATCACAGCTACGGTGACA	TGTGGTCCCGACTCTTCC	118
MBD4	8930	TGGAAGCTTCTCATCGCTAC	GCTGAAGGATACTTCTCCAGAAAC	95
MCL-1	4170	TGGGTTTGTGGAGTTCTTCCA	CTCCAGCAACACCTGCAAAA	83
MRE11A	4361	AGCCAGAGAAGCCTCTTGTA	TCTGGCTAAAGCGAAGAACAC	81
MSH2	4436	GCCCAGGATGCCATTGTTAA	TTGAGTGTCTGCATTGGTTCTAC	71
MSH3	4437	TGCCATTGCCTATGCTACAC	GGCGGATAATGGGTGACAAA	78
MSH6	2956	CCAAGGCGAAGAACCTCAAC	TTGGCCCAAACCAAATCTCC	97
NPM2	10361	AAGAAGATGCAGCCGGTCAC	GGGAGAAAGCTGCACTCCTAC	84
OOEP	441161	GTGCCTGGCATGGTTTCAC	TCTGATGCATGGGCCTTCAA	87
PADI6	353238	GTACGCCACAGTGAAGATGACA	ATCCTCGTTGGGCCCATAGTA	85
PCNT	5116	TCTCTGGAAGGTTTCAGCCG	CGCACCATCTGCAGTAAAGC	88
PDCD5	9141	GGCCCAACAGGAAGCAAA	ACTGATCCAGAACTTGGGCTA	71
PER1	5187	TCTGCCGTATCAGAGGAGGT	GGTCACATACGGGGTTAGGC	77
PLK1	5347	GCAGCGTGCAGATCAACTTC	CTCGTCGATGTAGGTCACGG	83
POT1	25913	CGAGGTAGAAAGATGTCAACAGCTA	CACATAGTGGTGTCCTCTCCAAA	77
POU5F1	5460	GCTTGGGCTCGAGAAGGATG	CATAGTCGCTGCTTGATCGC	80
PRMT1	3276	AGTTCACACGCTGCCACAA	GTCTGCTTCCAGTGCGTGTA	73
PTTG1	9232	GCCTCAGATGATGCCTATCCA	TCAGGCAGGTCAAAACTCTCA	80
RAD51	5888	GGGAAGACCCAGATCTGTCA	ATGTACATGGCCTTTCCTTCAC	86
RAD52	5893	GGATCTTGGGACCTCCAAACTTA	TCTTCATGTCCTGGCTCTTCC	85
RB1	5925	CTCACCTCCCATGTTGCTCA	GGGTGTTCGAGGTGAACCAT	73
RCC2	55920	TGCCTGTACCAAACGTGGTT	GGAGAAGACTCGCTTCTGGG	86
RPL10L	140801	CGAGGTGCCTTTGGAAAACC	TTCTGAAGCTTGGTGCGGAT	86
RPS24	6229	CGAATCGTGGTTCTCTTTTCCTC	TAGTGCGGATAGTTACGGTGTC	79
SMARCA4	6597	TCCGTGGTGAAGGTGTCTTA	AGCAAGACGTTGAACTTCCC	89
TERF1	7013	CAGCTTGCCAGTTGAGAACG	GGGCTGATTCCAAGGGTGTA	117
TERF2	7014	CCACCGTTCTCAACCAACC	AACCCCATTAGAGCTGTTCCA	80
TERT	7015	CGCCTGAGCTGTACTTTGTCA	TGATGATGCTGGCGATGACC	93
TP53	7157	GTGTGGTGGTGCCCTATGAG	CGCCCATGCAGGAACTGTTA	90
TSC2	7249	GCTGAACATCATCGAACGGC	CGTGGAACTCGTTCTGGTCA	122

TUBG1	7283	GGCTCATGATGGCCAACC	CGCTTACGCAGCTTGTCATA	79
XPA	7507	ACATCATTCACAATGGGGTGATA	ACCCCAAACTTCAAGAGACC	76
YBX2	51087	GTCCTGGGCACTGTCAAA	AAAGACATCTTCCTTGGTGTCA	81
YY1	7528	CCAAGAACAATAGCTTGCCCTCA	TGTTTTCTCATGGCCGAGTTATCC	71
ZAR1	326340	TGTGTGGTGTGTACAGGGAAC	TCCTCCACTCGGTAAGGGTT	90
ZSCAN4	201516	CCCGGGATTACCCAGTCAA	AGTCTCTTGCCTTGTGTCTCTA	91
ACTB	60	NA	NA	NA
CTNNB1	1499	NA	NA	NA
DDX4	54514	NA	NA	NA
GAPDH	2597	NA	NA	NA
GATA4	2626	NA	NA	NA
KHDC3L	154288	NA	NA	NA
MLH1	4292	NA	NA	NA
NLRP5	126206	NA	NA	NA
RPLP0	6175	NA	NA	NA
SOX2	6657	NA	NA	NA

NA, DNA sequence not available.
